# Preparation and Properties of Sulfur-Modified Alite Calcium Sulfoaluminate Cement

**DOI:** 10.3390/ma17246258

**Published:** 2024-12-21

**Authors:** Xiaodong Li, Guodong Kang, Shang Dou, Bing Ma, Jin Tang, Hao Zhou, Houhu Zhang, Jiaqing Wang, Xiaodong Shen

**Affiliations:** 1 Nanjing Institute of Environmental Sciences, Ministry of Ecology and Environment of the People’s Republic of China, Nanjing 210042, China; lixiaodong@nies.org (X.L.);; 2 State Key Laboratory of Materials-Oriented Chemical Engineering, College of Materials Science and Engineering, Nanjing Tech University, Nanjing 210009, China; 3 College of Civil Engineering, Nanjing Forestry University, Nanjing 210037, China

**Keywords:** C_4_A_3_$ and C_3_S formation, sulfur effects, mineral design, cement hydration

## Abstract

Alite calcium sulfoaluminate (ACSA) cement is an innovative and environmentally friendly cement compared to ordinary Portland cement (OPC). The synthesis and hydration of ACSA clinkers doped with gradient sulfur were investigated. The clinker compositions and hydrated pastes were characterized by X-ray diffraction (XRD), isothermal calorimetry, mercury intrusion porosimetry (MIP), and scanning electron microscopy (SEM) to analyze its mineral contents, hydration products, heat release, pore structure, and microstructure. The compressive strength and linear expansion of ACSA mortars were tested for their mechanical properties. Results showed that clinkers doped with 2 wt.% MgO can offset the hurdle that SO_3_ caused to the formation of C_3_S (tricalcium silicate). Clinkers with varying ratios of C_3_S and C_4_A_3_$ (calcium sulfoaluminate) were obtained, achieving 58–70% C_3_S and 2.0–5.6% C_4_A_3_$ in ACSA through adjusting the KH (lime saturation factor) values and SO_3_ dosage. ACSA cement showed better early mechanical properties. The 0.93 KH value with 3% SO_3_ dosage in the raw meal, which contains 63.9% C_3_S and 2.98% C_4_A_3_$ in the clinker, reached an optimal compressive strength level at 1d (26.35 MPa) and at 3d (39.41 MPa), marking 30.45% and 18.70% increases compared to PII 52.5. The excellent early strength of ACSA cement may offer promising applications t increasing the incorporation of supplementary cementitious materials, thereby reduce pollution and carbon emissions.

## 1. Introduction

The cement industry plays a vital role in modern construction [1]. In China, cement production was approximately 20.23 billion tons in 2023, which accounted for over 55% of global cement production [2]. Despite its widespread use, the process of cement manufacturing, particularly the sintering of limestone and clay, is a significant source of carbon dioxide (CO_2_) emissions [3], contributing around 26% of total global industrial emissions [2,4]. Thus, the cement industry is a focal point for low-carbon and emission reduction initiatives. There are three main approaches to achieving these goals [5]: (1) improving the durability of concrete during cement application to extend the lifespan of structures [6,7]; (2) enhancing the hydraulic properties of ordinary Portland cement (OPC) clinker to increase the use of supplementary cementitious materials (SCMs) [8,9]; and (3) developing innovative and environmentally friendly cement systems, such as calcined clay limestone cements (LC^3^) [10,11], calcium sulfoaluminate cement (CSA) [12,13], and alite–calcium sulfoaluminate cement (ACSA) [14,15].

ACSA cement is an energy-efficient cement that contains an appropriate amount of calcium sulfoaluminate (C_4_A_3_$) based on conventional Portland cement clinker. C_4_A_3_$ is a mineral with high early strength [16], which can decrease the shrinkage rate of traditional Portland cement to some extent and improve the physical properties of cement, such as frost resistance, impermeability, and durability [17]. ACSA cements may facilitate an increase in the dosage of supplementary cementitious materials (SMCs) and, thus, achieve the goal of pollution reduction and carbon emissions reduction. SMCs’ admixtures can reduce the early performance of cementitious systems [8]; hence, the primary focus of this paper regarding ACSA cement performance is the early strength. 

Alite forms abundantly at approximately 1450 °C, while C_4_A_3_$ decomposes almost completely at temperatures above 1350 °C. A protocol for the coexistence of both components was achieved by introducing a secondary high-temperature treatment after sintering, resulting in the formation of ACSA with good early strength [14]. In general, C_4_A_3_$ has great early strength performance, while C_3_S significantly contributes to the overall mechanical properties in ACSA cement. Therefore, studying the ratio of these two components is crucial for enhancing ACSA cement’s performance.

To investigate the effect of calcium silicate phases, previous work [18] has studied the effect of lime saturation factors (KH) on the synthesis and hydration behavior of ACSA clinker. Results showed that clinker with a KH of 0.93 has better mechanical strength. On the other hand, sulfur is an essential component for the formation of C_4_A_3_$ in the ACSA system. However, the dosage of sulfur in clinker can reduce the content of C_3_A and the value of C_3_S/C_2_S (dicalcium silicate) due to a decrease in the liquid phase’s viscosity and surface tension [19], hindering the formation of C_3_S and fostering a strong preference for C_2_S. Additionally, sulfur can impact the content of C_4_AF (brownmillerite). Therefore, MgO was added to compensate for the adverse effects of sulfur on the formation of C_3_S [20]. Moreover, the addition of a small amount of MgO and an alkali metal can decrease the sintering temperature, increase the liquid content, and reduce the viscosity of the liquid phase [21]. During the subsequent cooling process, most of the MgO remains in the aluminate and ferrite phases, and small amounts of MgO can disperse as small periclase crystals [22]. Studies have shown that the highest limit for the incorporated amounts of MgO in the clinker minerals is around 2 wt.% [20].

Some research has focused on the effects of KH, MgO, and sulfur on the sintering and mineral phase formation of ordinary Portland cement. However, the impact of MgO addition on the firing of ACSA cement, as well as the synergistic effects of KH and sulfur on the content of C_3_S and C_4_A_3_$ in the ACSA cement and the performance of ACSA cement, have not yet been thoroughly investigated. At the same time, regulating the contents of KH, MgO, and sulfur is of significant importance for the optimization of mineral phases in ACSA clinker and the enhancement of its performance.

In this work, raw meals with and without a 2 wt.% dosage of MgO were annealed along a gradient of temperatures to evaluate MgO effect on clinker sintering. Meanwhile, the clinker composition evolution over KH and sulfur was studied to determine the optimal level of C_3_S and C_4_A_3_$ content. The hydration properties of different contents of sulfur with a KH of 0.93 were studied to compare their strength performance. In summary, this research offers significant understanding into the production and hydration processes of ACSA cement, emphasizing the necessity of refining its composition to optimize its mechanical properties.

## 2. Materials and Methods

### 2.1. Sample Preparation

Industrial raw materials, including industrial limestone (from China United Concrete Nanjing Co., Ltd., Nanjing, China), first class fly ash, clay, and silica, were ground to pass through an 80 μm sieve. All chemical reagents, including MgO, K_2_O, and CaSO_4_·2H_2_O, were purchased from Sigma–Aldrich (St. Louis, MO, USA) and used as received. Chemical compositions of the raw meals are shown in Table 1. The sintering protocol of ACSA clinker has been reported in previous work [18]. A secondary heat treatment at 1270 °C for 1 h, after ordinary sintering at 1470 °C and fast cooling at room temperature, was conducted to achieve the coexistence of alite and ye’elimite [10,18]. The raw meals of M0 and M2 were taken out at 900, 1000, 1100, 1200, 1300, 1400, and 1500 °C, respectively, to evaluate the effect of MgO on clinker sintering. A tungsten carbide vibrating mill was used for two cycles of 10 s each to grind the clinker blocks into fine powders. ACSA cements were prepared by blending the described clinker powders with 4 wt.% of gypsum [18].

### 2.2. Characterization

#### 2.2.1. X-Ray Diffraction and Rietveld Quantitative Analysis

XRD was performed using a Rigaku Miniflex workstation (Miniflex 600, Rigaku Co., Tokyo, Japan) operating at 40 kV and 15 mA with Cu Kα radiation (λ = 0.1789 nm). Data were collected from 5 °C to 70 °C (2θ) for 13 min with a step size of 0.01 °C. Rietveld [23] quantitative analysis was conducted using GSAS EXPGUI 1.0 version [24] with crystal structures for refinement, including C_3_S (alite, ICSD 64759), C_2_S (larnite, ICSD 79553), C_4_AF (brownmillerite, ICSD 2841), C_3_A (ICSD 1841), C_4_A_3_$ (ye’elimite ICSD 9560), and f-CaO (free lime, ICSD 52783). The background and profile fittings used the shifted Chebyshev polynomial and pseudo-Voigt functions, refining phase fractions, lattice parameters, and profiles (GU, GV, and GW).

#### 2.2.2. Microscopy

Clinker granules were embedded in two-component epoxy, polished successively with sandpaper with a 600, 1200, 3000, and 5000 mesh, and etched with a 1% HNO_3_ alcohol solution for 10 s to prepare the surface [25]. Microscopic images were captured using a Leica Microsystems GmbH microscope (Wetzlar, Germany) equipped with a Leica DFC 480 camera.

#### 2.2.3. Particle Size

The clinker powders’ particle size distribution (PSD) was measured with Malvern MS2000 laser diffraction granulometry (Malvern Panalytical, Spectris, London, UK) after dispersing in ethanol via ultrasonication for 60 s, with reflection indexes of 1.70 and 1.36 for the clinker and ethanol, respectively.

#### 2.2.4. Compressive Strength Tests

Mortars were prepared in accordance with the Chinese National Standard (GB/T 1346 2011) [26], with a weight ratio of ACSA cement/sand/water = 1/3/0.5. The mortars were placed in a mold (40 × 40 × 160 mm) at 20 ± 1 °C and 95% relative humidity for 24 h. Subsequently, the mortars were removed from the mold and immersed in water at 20 °C until they were ready for strength testing. The compressive strengths of the mortars were tested according to GB/T17671-2021 [27] using an automatic cement strength testing machine (AEC 201, Ruifeng, Shanghai, China) with a maximum load of 200 kN and a constant loading rate of 2.4 kN/s. The average of four tested specimens represented each compressive strength value.

#### 2.2.5. Expansion Tests

Pastes were prepared in accordance with the Chinese National Standard (GB/T 1346 2011) [26]. Samples were added to 4% dihydrate gypsum with a water–cement ratio of 0.35 and set in a 20 × 20 × 80 mm mold. The length of each age was tested, and the expansion is represented by the change rate of the duration relative to the base length. Each sample was formed to 6 pieces and tested averaged.

#### 2.2.6. Calorimetry

Isothermal calorimetry was conducted at 20 °C for 72 h using an eight-channel thermometric air instrument (TAM Air Isothermal Calorimeter, TA Instruments, New Castle, USA) to measure the hydration heat release of pastes. Pastes were prepared with a w/c ratio of 0.5 in plastic ampoules that were externally mixed prior to the test.

#### 2.2.7. Mercury Intrusion Porosimetry (MIP)

Porosity and pore size distribution of hydration pastes were characterized using a Poremaster GT-60 (Quantachrome, Boynton Beach, FL, USA) through mercury intrusion porosimetry (MIP). Regularly shaped, cracked pieces that were not in contact with the mold were chosen for the measurement.

#### 2.2.8. SEM

The microstructures of hydrated pastes were observed using a scanning electron microscope (SEM, Model JSM-5900, JEOL Co., Tokyo, Japan) after fracturing the specimens and attaching them to the specimen holder with carbon tapes. A thin layer of gold was then applied to the specimens to enhance electrical conductivity.

## 3. Results and Discussion

### 3.1. Clinker Sintering

#### 3.1.1. Burnability of the Sulfur-Containing Clinker

**Figure 1:**
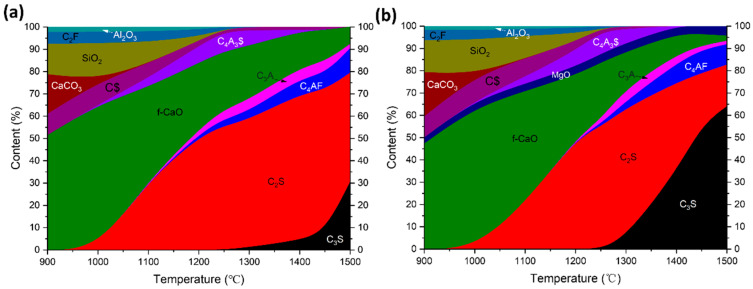
Formation of the clinker during the sintering process: (**a**) M0 and (**b**) M2.

The evolution of mineral compositions calculated by the Rietveld [23] method from the XRD data is shown in Figure 1. C_3_S in sample M2 formed in large quantities after 1300 °C (Figure 1b), which is about 100 °C lower than that of the M0 sample (Figure 1a). Moreover, at 1500 °C, the content of C_3_S in the M2 sample is about 65%, which is more than double the C_3_S content in M0. It is obvious that the MgO dosage can effectively promote the formation of C_3_S in the sulfur-containing clinker. The sulfur in the solid solution of C_2_S is four to five times that of C_3_S, which makes C_2_S in sulfur-containing clinker more stable, thus hindering the formation of C_3_S [19]. The addition of MgO can reduce SO_3_ content in the solid solution of C_2_S, and reduce the obstacle of sulfur to the formation of C_3_S [19]. C_4_A_3_$ formed in large quantities after 1250 °C and vanished after 1400 °C, indicating the necessity of the secondary heat treatment to achieve the coexistence of C_3_S and C_4_A_3_$ [14].

#### 3.1.2. Mineral Compositions of the Clinkers

**Figure 2:**
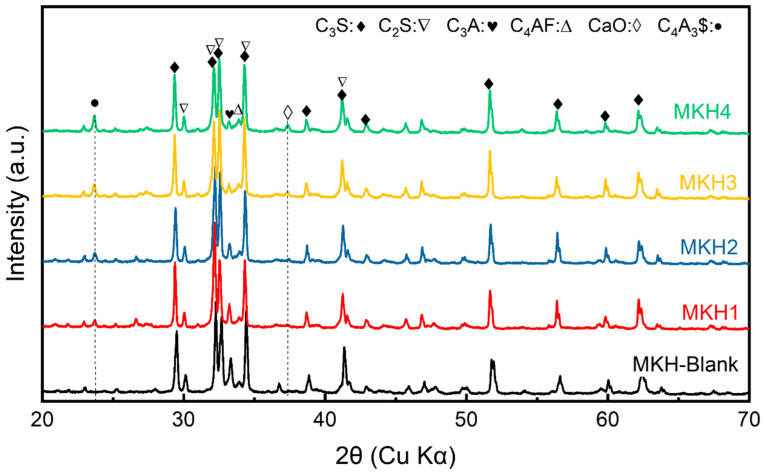
XRD spectra of clinkers with different SO_3_ dosage.

**Figure 3:**
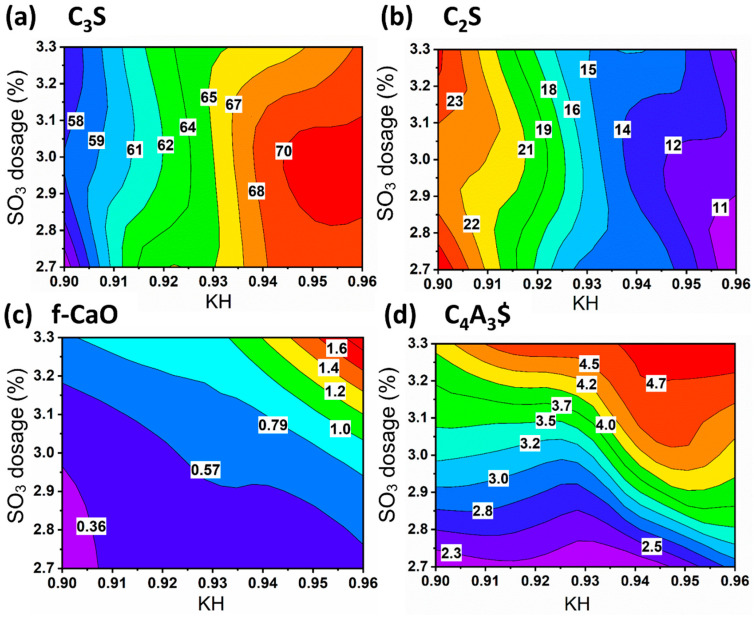
Contour of mineral compositions as a function of KH values and SO_3_ content: (**a**) C_3_S, (**b**) C_2_S, (**c**) f-CaO, and (**d**) C_4_A_3_$.

The XRD spectra, the mineral composition calculated by the Rietveld method, and the contour based on the quantitative results of the clinkers are illustrated in Figure 2, Table 2, and Figure 3, respectively. The peaks of the C_4_A_3_$ fingerprints at 23.5 °C 2θ on the XRD spectrum gradually enhance with an increase in SO_3_ content, while the characteristic peak of C_3_A decreases (Figure 2), indicating the formation of C_4_A_3_$ from C_3_A and SO_3_.

The fingerprint of f-CaO at 37.5 °C 2θ increases with the SO_3_ content [28]. At the same time, the increase in SO_3_ content leads to a decrease in the C_3_S/C_2_S ratio. Belite (C_2_S) has a much stronger ability to incorporate sulfur than alite (C_3_S), making sulfur-containing belite more stable [19]. This stability inhibits the reaction of C_2_S with CaO to form C_3_S (C_2_S + CaO → C_3_S), thereby hindering the formation of C_3_S in the clinker and resulting in more f-CaO in the clinker. In addition, after SO_3_ dosage, the fingerprint of C_3_S in the 51.2–52.4 °C 2θ region of the two peaks of about the same height changes to one peak with a shoulder, indicating the crystalline transformation of M3 to M1 [29].

Figure 3 shows the contour plots of various minerals as a function of KH values and SO_3_ content. Figure 3a demonstrates that the formation of C_3_S shifts to the right, indicating that KH values play a decisive role in the formation of C_3_S. However, when KH values reach a certain level, SO_3_ begins to hinder the formation of C_3_S. The trend in C_2_S is opposite to that in C_3_S, which is consistent with our expectations. For f-CaO and C_4_A_3_$, Figure 3c,d both show an upward-rightward inclination. It is important to note that the content of f-CaO should not be too high, as it needs to ensure the clinker quality while achieving the formation of C_4_A_3_$ minerals.

#### 3.1.3. Mineral Microscopy of the Clinkers

**Figure 4:**
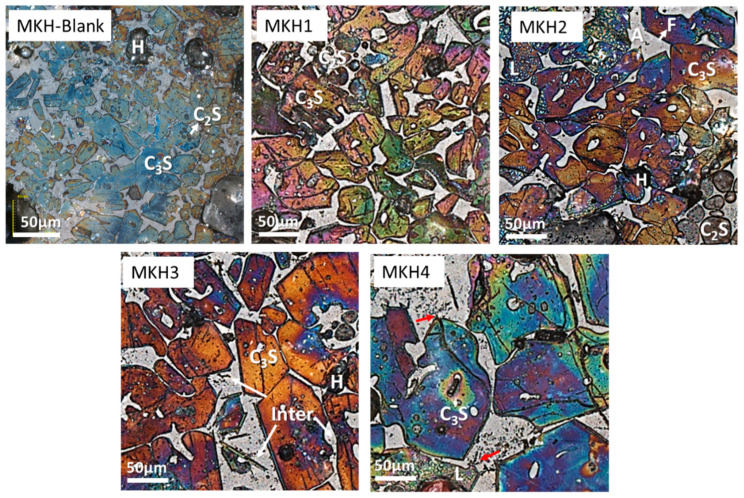
Microscopy images of clinkers. C_3_A is alite, C_2_S is belite, A is aluminate, F is ferrite, L is free lime, H is hole, and Inter. denotes interstitial phases.

The optical microscope images of the samples show that, in the blank sample, most of the C_3_S appears as small hexagonal platelets. The addition of SO_3_ promotes the growth of large C_3_S grains, with the MKH4 sample exhibiting grains exceeding 100 μm in size. Furthermore, with SO_3_ doping, most of the C_3_S exhibits hexagonal or prismatic shapes with distinct grain boundaries. On the contrary, C_2_S consists mostly of small circular grains, contributing relatively less to the overall composition, consistent with quantitative XRD analysis results. Bubble-like free lime is located at the boundaries of C_3_S (Figure 4). Meanwhile, the intermediate phases of all clinkers are finely distributed, indicating a moderate liquid phase content and good burning. The brighter and grey phases intermingled in intermediate phases are ferrite and aluminate (F and A in Figure 4). Some samples show slight black speckled stripes, which are speculated from previous results to be C_4_A_3_$ [18,30].

### 3.2. Mortar Properties

#### 3.2.1. Compressive Strength

**Figure 5:**
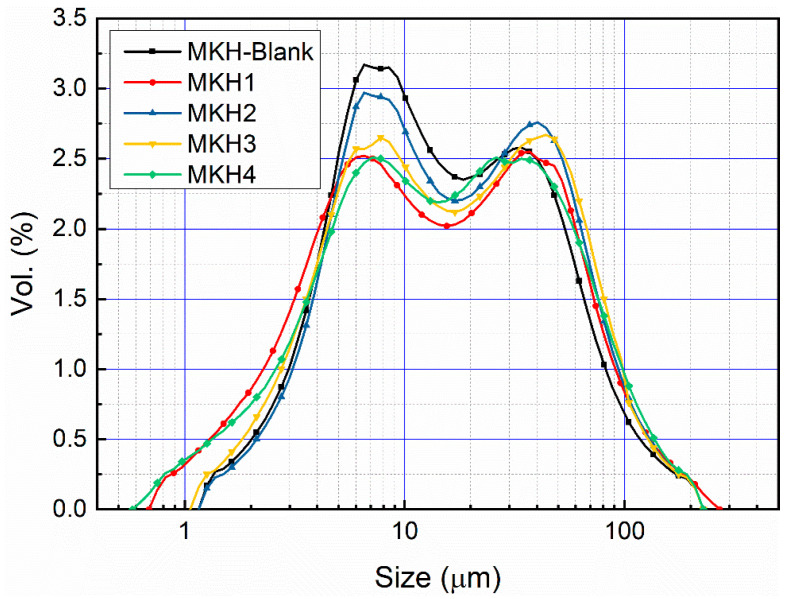
Particle size distribution of ACSA clinkers for mechanical strength measurement.

Clinkers of varying SO_3_ levels were ground using a uniform protocol, yielding particle size distributions with two peaks at 5–10 and 20–30 μm (Figure 5). These powders were then utilized to assess mortar compressive strength and paste hydration characteristics; owing to their similar particle size distribution, their impact on cement performance can be considered negligible.

**Figure 6:**
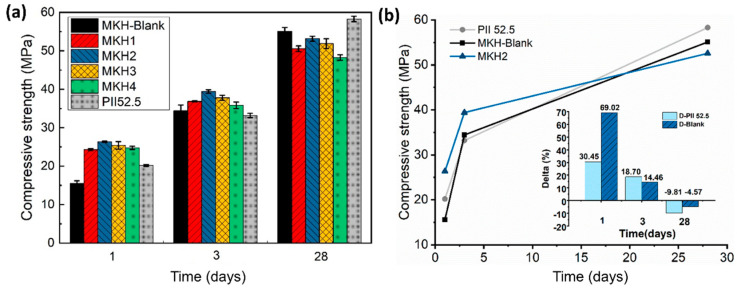
Compressive strength of all the mortars (**a**), compressive strength comparison among PII 52.5, MKH-Blank, and MKH2 (**b**). The inset plot in (**b**) represents the compressive strength increment of MKH2 compared to MKH-Blank and PII 52.5. For D-Blank, Delta = (MKH2 − MKH-Blank)/MKH-Blank; for D-PII 52.5, Delta = (MKH2 − PII 52.5)/PII 52.5.

The compressive strength results for the 1-, 3-, and 28-day curing ages of the samples with the same KH (0.93) but different C_4_A_3_$ contents are shown in Figure 6. The compressive strength of 1-,3-day curing ages increased significantly due to the existence of C_4_A_3_$ (MKH1~MKH4). However, an increase in C_4_A_3_$ content did not necessarily correspond to an increase in clinker strength. Excess C_4_A_3_$ content can severely affect the later strength of the clinker. For example, MKH4 (with a 5.61% C_4_A_3_$ content) exhibits a later strength of only 48.26 MPa, a decrease of nearly 7 MPa compared to the blank sample. Furthermore, for both 1-day and 3-day strengths, a higher C_4_A_3_$ content did not necessarily result in a better performance; instead, an intermediate value tended to yield optimal results. MKH2 exhibited the best overall strength performance: 26.35 MPa at 1 d, 39.41 MPa at 3 d, and 53.15 MPa at 28 d, with 30.45% and 69.02% increases in 1-day strength and 18.70% and 14.46% increases in 3-day strength compared to PII 52.5 and MKH-Blank (Figure 6b). Meanwhile, the compressive strength of the 28-day sample was only decreased by 1.95 MPa compared with MKH-Blank.

#### 3.2.2. Expansion Performance

**Figure 7:**
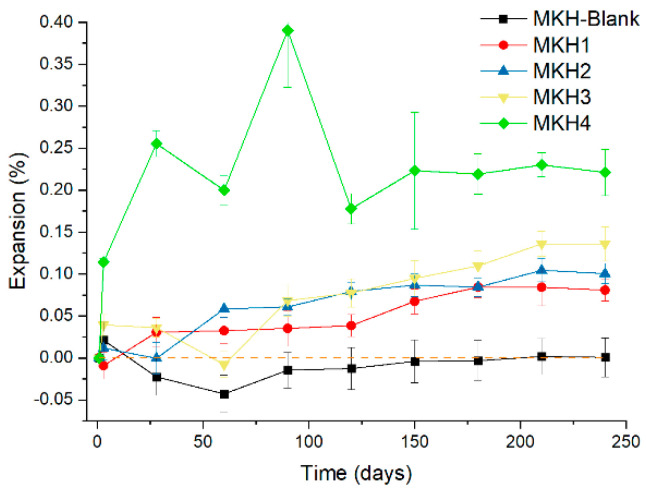
Linear expansion of the mortars.

The linear expansion value of the MHK-Blank sample stayed below 0 (Figure 7). The expansion in cement paste increased with the C_4_A_3_$ content, which corresponds to the formation of ettringite (C_6_A$_3_H_32_, AFt) from C_4_A_3_$ hydration [17]. Apart from MKH4, expansion in the pastes was below 0.1% within 150 days and converged below 0.15% by around 200 days. However, for the MKH4 sample, its expansion was much higher—about two times that of the other samples—and this higher expansion rate may also be the reason for the poorer development of its strength. This expansion property of ACSC cement may be a method of reducing shrinkage and cracking in cement-based materials [31].

### 3.3. Analysis of Hydration Products

#### 3.3.1. Heat Release of Hydration

**Figure 8:**
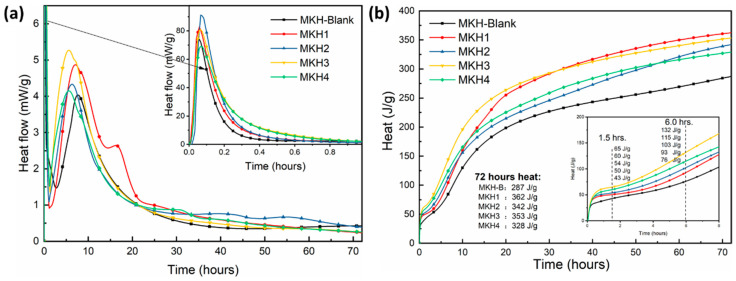
Hydration heat evolution of the clinkers: heat flow (**a**) and accumulative heat (**b**). The inset plot in (**a**) represents the heat release during clinker dissolution within the first hour of hydration.

In the Portland cement system, the C_3_A and C_3_S minerals are involved in early hydration. Whereas, in this matrix, calcium sulfoaluminate reacts with gypsum to form ettringite (C_6_A$_3_H_32_, AFt), C_4_A_3_$ + 2C$H_2_ + 34H → C_6_A$_3_H_32_ + 2AH_3_, releasing a significant amount of heat during early hydration. Figure 8a shows the hydration heat evolution curve of the MKH group clinkers over 72 h, with all samples containing 4% dihydrate gypsum. The main exothermic peak is attributed to the hydration of C_3_S. The position of this peak shifts to an earlier time with an increase in C_4_A_3_$ content, indicating a shorter induction period that is influenced by sulfur content [17]. While the inset depicts the heat release peak when cement samples are in contact with water, primarily from the particle dissolution and the hydration of C_3_A and C_4_A_3_$, the intensity of this peak first increases and then decreases with an increase in C_4_A_3_$ content. A shoulder peak is obviously observed on MKH2 within 15–20 h of hydration, which corresponds to the transformation of monosulfate (C_3_A$H_12_, AFm) from AFt [32].

The cumulative heat release of MKH group cement samples is shown in Figure 8b, with the inset illustrating cumulative heat release within 8 h of hydration. Within this timeframe, except for sample MKH4, the cumulative heat release correlates positively with the content of C_4_A_3_$; for instance, at 6 h, the heat released by the MKH-Blank, MKH1, MKH2, and MKH3 samples are 76 J/g, 93 J/g, 103 J/g, and 132 J/g, respectively. Regarding the total heat release over 72 h, samples containing C_4_A_3_$ exhibited significantly higher— at least 50 J/g higher—values compared to the MKH-Blank.

#### 3.3.2. Mineral Composition of the Pastes

**Figure 9:**
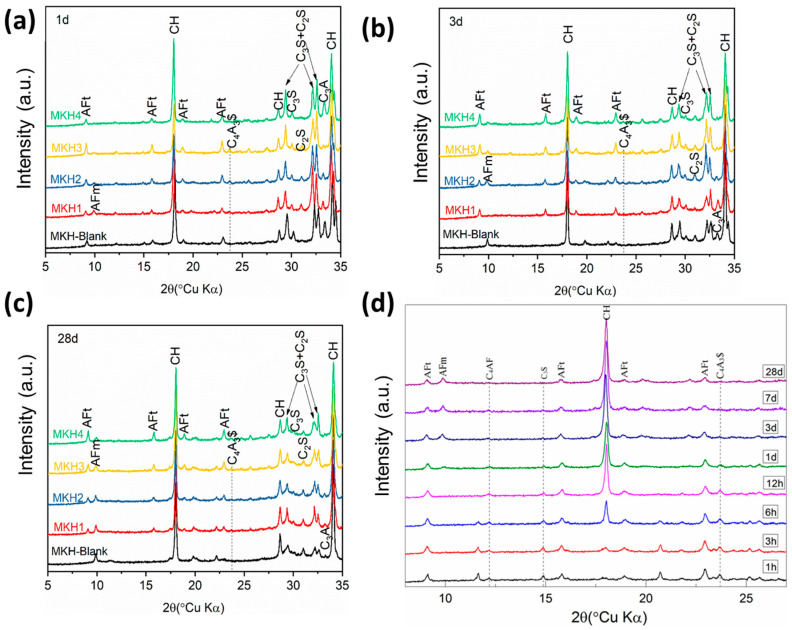
XRD spectra of the hydration pastes at (**a**) 1, (**b**) 3, and (**c**) 28 d. XRD spectra of hydrated MKH2 from 1 h to 28 d (**d**).

XRD analysis was used to characterize the hydration products (Figure 9). The hydration products primarily included calcium hydroxide (CH), AFt, and AFm. The CH mainly originated from the hydration of C_3_S, and its presence can enhance the hydration of C_4_A_3_$ [30]. C_4_A_3_$ underwent hydration at 1 d to form Aft, and almost all of the C_4_A_3_$ completed its reaction by 3 d. With the consumption of sulfur, the AFt gradually transformed into AFm. MKH1 shows the highest content of AFm at 1 d (Figure 9a), which is consistent with the heat release results (Figure 8a). In MKH-Blank, AFt completely transformed into AFm by 3 d, while AFt can be observed in the C_4_A_3_$-containing samples until 28 d. Figure 9d illustrates through the evolution of hydration products over time that CH begins to form significantly between 3 and 6 h after cement hydration starts, while AFt forms in large quantities right at the beginning of hydration. AFm formation occurs later, starting around 3 d of hydration, whereas C_4_A_3_$ has completely reacted by this period.

#### 3.3.3. Porosity

**Figure 10:**
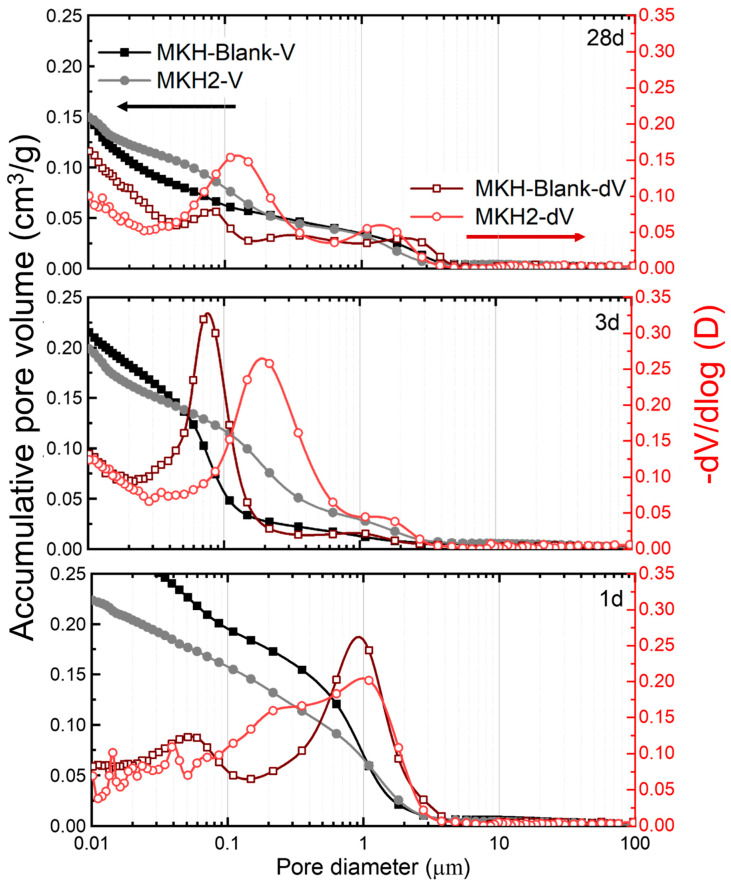
Pore volume and pore size distribution of the hydrated pastes.

Due to the presence of the C_4_A_3_$ mineral, the alite calcium sulfoaluminate cement prepared in this experiment exhibited slight early-stage expansion characteristics and formed a large amount of AFt during early hydration. This compensated for its shrinkage during cement hydration and, to some extent, served as a filler. Figure 10 and Table 3 show the development of the pore structure of samples with and without the C_4_A_3_$ mineral at various ages of hydration. In the early stages of cement hydration, the total pore volume of MKH2 was significantly smaller than that of MKH-Blank. At 1 day of hydration, the porosity of MKH-Blank was 40.95%, while MKH2’s was 34.17%, showing a reduction of 6.78%. This is a major reason for the early development of strength in C_4_A_3_$-containing cement. The difference in porosity between the two samples gradually decreased over hydration time, making a tendency for their compressive strengths to be consistent (Figure 6). Figure 10 also illustrates the pore size distribution of the cement paste. It shows that, with increasing hydration age, the large pores decrease and the small pores increase in both the MKH-Blank and MKH2 hardened pastes. MKH2 shows a wide pore size distribution at 1 d, a narrow distribution with an obvious peak around 200 nm at 3 d, and two separated peaks at 28 d. While the pattern of pore size distribution of MKH-Blank shows the opposite trend.

#### 3.3.4. Microstructure

**Figure 11:**
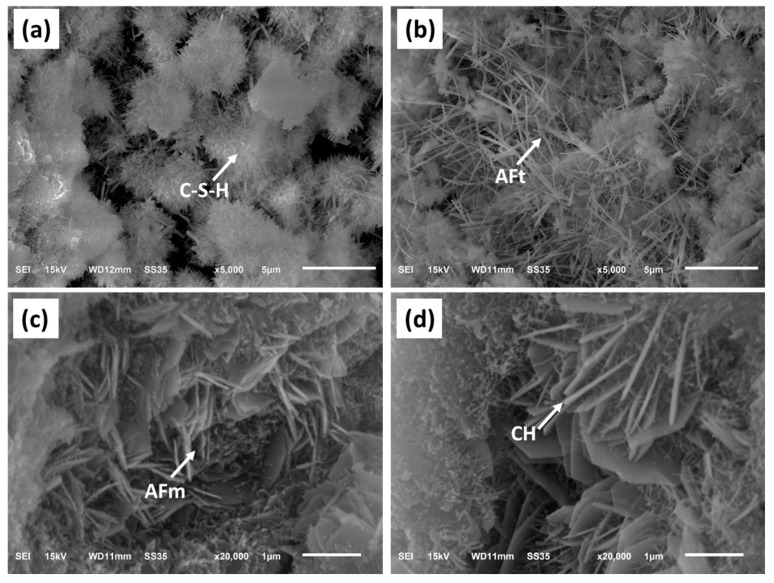
SEM of (**a**) MKH0-1d, (**b**) MKH2-1d, (**c**) MKH0-28d, and (**d**) MKH2-28d.

Typical needle-shaped AFt [33] in the C_4_A_3_$-containing sample can be observed in the SEM images, with fine and elongated crystals at 1 day (Figure 11b). Due to the early hydration product of C_3_S being a C-S-H gel, this enhances the bonding between AFt and other hydration products [30]. The needle-shaped AFt crystals also serve as a framework of support in the C-S-H gel, explaining why the compressive strength of the MKH2 samples at 1 day is significantly higher than that of MKH-Blank. Figure 11c also confirms the presence of petal-like AFm [34], which is in accordance with the mineral composition results (Figure 9c).

## 4. Conclusions

A great amount of C_3_S and C_4_A_3_$ could coexist in a burning system with a sintering temperature of 1470 °C and a secondary heat treatment temperature of 1270 °C, while the content of f-CaO is within a desirable range. Clinkers doped with 2 wt.% MgO can offset the hurdle that SO_3_ may cause to the formation of C_3_S. Through adjusting the KH values (0.90–0.96) and SO_3_ dosage (2.4–3.3 wt.%) in clinker preparation, clinkers with varying ratios of C_3_S and C_4_A_3_$ were obtained, achieving 58–70% C_3_S and 2.0–5.6% C_4_A_3_$ in ACSA clinkers. Based on the above experimental results and discussion, the following conclusions can be drawn:

Due to the early hydration formation of abundant AFt by calcium sulfoaluminate, ACSA cement shows a higher hydration heat release, with a 72-hour heat release that is 50–70 J/g higher than the blank sample.

C_4_A_3_$ is almost completely reacted within 3 days of hydration, accompanied by gradual AFm formation. Meanwhile, ACSA cement shows better early mechanical properties.

The 0.93 KH value with 3% SO_3_ dosage in the raw meal (sample MKH2), which contains 63.9% C_3_S and 2.98% C_4_A_3_$ in the clinker, reaches an optimal compressive strength level: at 1 d (26.35 MPa) and at 3 d (39.41 MPa), marking 30.45% and 18.70% increases compared to PII 52.5.

Because of the higher early age strength of ACSA cement, this cement has the potential to increase the utilization of supplementary cementitious materials, thereby reducing pollution and carbon emissions. Furthermore, the controlled expansion characteristics of ACSA cement may decrease shrinkage and cracking phenomena in cementitious materials, consequently enhancing the service life and durability of concrete structures.

Although it is possible to achieve the coexistence of the difficult-to-coexist C_3_S and C_4_A_3_$ at a firing temperature of 1470 °C and a secondary heat treatment temperature of 1270 °C, this condition is not easy to realize in actual cement production. Future research should focus on exploring conditions at a temperature between 1270 °C and 1470 °C to achieve the coexistence of C_3_S and C_4_A_3_$ through a single firing process. This study has significantly improved the early strength of ACSA cement by regulating KH values and the dosage of SO_3_, and it has explained the reasons behind this enhancement in early strength. However, the factors affecting the strength of ACSA are not limited to the contents of C_3_S and C_4_A_3_$, as well as the regulation of KH values and SO_3_ dosage. Future research should focus on other factors, such as the synergistic optimization of mineral contents and the optimization of ACSA cement particle size, to achieve superior performance in the 28 d strength of ACSA cement.

## Figures and Tables

**Table 1:** Chemical compositions of the raw meals (wt.%).

Labels	CaO	SiO_2_	Al_2_O_3_	Fe_2_O_3_	MgO *	K_2_O *	SO_3_ *	KH **	SM **	IM **
MKH-Blank	66.27	21.53	5.98	1.88	2.00	0.80	0.00	0.93	2.50	3.10
MKH-1	65.11	20.03	5.62	1.76	2.00	0.80	2.40	0.93	2.50	3.10
MKH-2	63.97	20.11	5.66	1.71	2.00	0.80	2.70	0.93	2.50	3.10
MKH-3	64.22	20.12	5.62	1.70	2.00	0.80	3.00	0.93	2.50	3.10
MKH-4	64.22	20.12	5.62	1.70	2.00	0.80	3.30	0.93	2.50	3.10
LKH-2	64.62	20.42	5.62	1.81	2.00	0.80	2.70	0.90	2.50	3.10
HKH-2	65.72	19.78	5.50	1.77	2.00	0.80	2.70	0.96	2.50	3.10
M0	64.22	20.12	5.62	1.70	0	0.80	3.00	0.93	2.50	3.10
M2	64.22	20.12	5.62	1.70	2.00	0.80	3.00	0.93	2.50	3.10

* The weights of MgO, K_2_O, and SO_3_ were calculated as exogenous ingredients in the raw meal. ** Lime saturation factor (KH) = (CaO − 1.65 A1_2_O_3_ − 0.35 Fe_2_O_3_)/(2.8 SiO_2_), silica modulus (SM) = SiO_2_/(Al_2_O_3_ + Fe_2_O_3_), and iron modulus (IM) = Al_2_O_3_/Fe_2_O_3_ [19].

**Table 2:** Mineral compositions of the clinkers calculated using the Rietveld method (%).

Samples	C_3_S	C_2_S	C_3_A	C_4_AF	f-CaO	f-MgO	C_4_A_3_$	R_wp_
MKH-Blank	68.07	12.94	11.68	4	0.02	3.09	0	8.63
MKH1	63.74	18.17	7.54	4.51	0.51	3.38	2.02	11.87
MKH2	63.90	18.55	5.96	4.57	0.58	3.27	2.98	11.07
MKH3	64.36	18.18	3.32	5.64	0.72	3.27	4.52	10.00
MKH4	64.65	17.62	2.42	4.86	1.38	3.14	5.61	8.51
LKH2	58.23	23.65	6.84	4.61	0	3.58	2.88	8.31
HKH2	70.63	11.69	6.47	4.56	0.50	3.28	3.26	10.81

**Table 3:** Total porosity (%) and the pore volume (cm^3^/g) of the hydrated pastes.

Samples	Porosity (%)			Pore Volume (cm^3^/g)		
	1 Day	3 Day	28 Day	1 Day	3 Day	28 Day
MKH-Blank	40.95	34.00	24.23	0.2968	0.2319	0.1614
MKH2	34.17	31.25	24.42	0.2217	0.2134	0.1586

## Data Availability

The original contributions presented in this study are included in the article; further inquiries can be directed to the corresponding authors.

## Nomenclature

C = CaO, S = SiO_2_, A = Al_2_O_3_, F = Fe_2_O_3_, $ = SO_3_, nomenclature is not suitable if the compounds are described in their full representation [32].
